# Supplementary material for: Hospital care does not meet the communication needs of patients with hearing loss: A qualitative study of patient experiences
Source: PLoS One. 2025 Oct 10;20(10):e0333587. doi: 10.1371/journal.pone.0333587 (PMC12513649; doi:10.1371/journal.pone.0333587)
Supplement: S1 File — (DOCX) [file pone.0333587.s001.docx]

**Focus Group and Interview guides**

**Focus group guide**

1. When you were in the hospital, what was your experience communicating with healthcare providers?
2. Did your healthcare team know that you have difficulty hearing?
3. What would have made your experience communicating with others in the hospital better?

**Interview guide**

1. Tell me about your experiences with communication in the hospital.
2. Do your healthcare providers here know that you have hearing difficulties?
3. Have your providers used any communication tools like a microphone or writing things down?
4. What makes communication harder for you in hospital?
5. Do you have to tell every new provider that you have hearing difficulties?
6. How do you think hearing loss affects your experience at the hospital?
7. What would you change if you could?
8. What would you like your providers to know?
