# Supplementary material for: Hospital care does not meet the communication needs of patients with hearing loss: A qualitative study of patient experiences
Source: PLoS One. 2025 Oct 10;20(10):e0333587. doi: 10.1371/journal.pone.0333587 (PMC12513649; doi:10.1371/journal.pone.0333587)
Supplement: S2 Table — (DOCX) [file pone.0333587.s002.docx]

**Table 2.** **Additional participant quotes to support each theme and subtheme**

| **Theme** | **Example quote** |
| --- | --- |
| **Theme 1: Hearing loss is an invisible disability** | |
| Hearing loss is often overlooked in healthcare | *“Hearing [loss] is like an invisible disease because nobody sees it.”* – Participant 13 |
|  | *“If you have an invisible disability, you're ignored, by and large.”* – Participant 3 |
|  | *“I'm not stupid, I just can't hear properly.” –* Participant 14 |
|  | *“Certainly it's not on [medical] charts, it's not on your day-to-day care plan, and it's not even a priority because most hearing loss is disregarded and misdiagnosed.”* – Participant 4 |
|  | *“I had it on my files that I'm hard of hearing/deaf [but] medical professionals don't read that information”* – Participant 10 |
|  | *“My experience at the time of my implant surgery was not positive because my husband was the one that ended up doing all [communicating] for me, and so I felt like I was just sort of lying there kind of useless while communication was attempted sort of across from me” –* Participant 11 |
| Communication should be equitable to all patients | *“If you're having troubles with your kidneys or liver or pancreas or whatever that are life threatening, you deserve the same sort of treatment as somebody with a heart problem. And if you are hard of hearing, there needs to be accommodation for that because it is becoming more and more of a problem.”* – Participant 3 |
|  | *“People don’t see it equivalent to other disabilities. Yes, it’s an invisible disability but like, as we were discussing … we shouldn’t really have to fully identify ourselves. Because it’s like, if somebody is in a wheelchair and they need an elevator, they don’t have to announce “hey I’m going to go be using the elevator now”, they just wheel up and use it. If they have to use accessible bathrooms, it’s not announced to anybody, they just go and use it.”* – Participant 10 |
|  | *“Well what I find shocking is hospitals or other facilities, they say “we provide interpreters for 200 languages”, but they won’t provide some sort of either interpreter or listening device that can accommodate the hard of hearing in English.”* – Participant 10 |
|  | *“I think if you have really good hearing you have a conversation with the medical personal, but if you have a hearing aid, it seems like you, it’s like doctors just ignore them.”* – Participant 12 |
|  | *“It is absolutely essential that patients have full understanding of what is going on as opposed to ‘we got the gist of it’.”* – Participant 2 |
|  | *“It's about respect and non-judgment on the part of the healthcare professional. I have had times where it almost feels like the person is getting frustrated because I can't hear what they are saying and it is not like it's my fault that I [have hearing loss] … so being able to go to the hospital … and know that I would be accommodated, my needs would be accommodated … having that comfort in knowing I would be treated with respect and have that accommodation that I needed.”* – Participant 4 |
| **Theme 2: Communication is a team effort** | |
| Patients must be encouraged and supported to disclose their communication needs | *“I think there is denial or there is a stigma I think attached to it which I think because we're here we don't care, we will tell people we have a hearing loss, but I think there are a lot of people who want it confidential and so a lot of these things maybe wouldn't work because we have to go through that stage of getting over ourselves and saying, yes, I have this hearing loss, and I need some help.”* – Participant 6 |
|  | *“So it is not only a one-way street for the hard of hearing that need the help, the medical professional part need the help too, and that may be in more ways than I would like to elaborate but it would make things go so much smoother and save a lot of funding in my opinion.”* – Participant 10 |
|  | *“I point to my ears and make a gesture with my hands to let them know. And sometimes I just say – my hearing aids are out, wait until I put them in.”* – Participant 14 |
|  | “*Because the first thing I do, I would just tell them, if I go to the emergency room, I have a cochlear implant, so I will tell them up front, I'm sorry, I have a cochlear implant, and then no problem, we proceed to next steps.”* – Participant 7 |
|  | *“You can't expect healthcare to know all of you … you have to be able to speak up a little bit for yourself. But there are people there that won't do that. They're not comfortable or don't think of it. So that's hard to fix.” –* Participant 1 |
|  | *“Most people who have [hearing loss] know they’ve got it, but they may not tell you.” –* Participant 2 |
| Providers need to accommodate | *“I feel especially in healthcare I'm more self-conscious about asking for that support from the receptionist or from a nurse. I am also part of the LGBTQ2S+ community, and I know that when I see Pride flags in a pharmacy or doctor's office, I feel safe in that space. Maybe even just a disability flag [would help], maybe it is that simple.” –* Participant 8 |
|  | *“If [hearing loss] could be incorporated into the paperwork somehow and get passed on to your treatment team … make sure this patient understands what you are saying because there is a hearing [loss] here.” –* Participant 14 |
|  | *“I think there are numerous places where [hearing loss] could be identified. I wear my badge most of the time … it says "please speak clearly, I'm a lipreader" … I have read some literature that suggests it is going to have to be in multiple places. On the chart is only one, but there is so many sections to a chart that everybody who sees you is not going to be looking at it. So, what's wrong with having it at the end of the bed, what's wrong with having it on a bracelet if you can't speak.” –* Participant 5 |
|  | *“I know that [healthcare providers] are busy … if they know that they are dealing with somebody who is deaf or has difficulty with hearing … either they have something they can communicate voice-to-text … or a white board … or just slowing speech down … not turning your back to somebody … the face-to-face is important.”* – Participant 11 |
|  | *“I went into the hospital … I asked ‘do you have a Pocketalker?’ and they proceeded to say ‘Yes, we do … that's in the speech area … [it’s] closed and locked up. We can't access that until tomorrow morning’. Well, my appointment is now, so that's not going to help me. So I think whatever is put in place for accommodations needs to be accessible at any time and no matter what department or area you are going to.”* – Participant 10 |
|  | *“Because we can't hear, we have more adverse outcomes. We know that hearing loss causes falls, so that extends the hospital stay or complicates it, re-admission is 30% [higher] and this is in a population of over 70 years of age. So administration needs to be educated about this as well in order to support the staffing and the policy-makers and so on.”* – Participant 5 |
| **Theme 3: Every patient has different needs in different situations** | |
| People have different needs and preferences for communication solutions | *“Even knowing things like there are different types of hearing loss and different things work for other people. I have conductive loss, and, honestly, when people speak really loud, that's great for me. But conductive loss is not the most common kind of loss. So most people don't want you to speak loud. That's not helpful to them. But it is to me. And knowing that just because something worked for one person may not work for someone else it would be useful when they first contact people to know”* – Participant 2 |
|  | *“Everybody is different at different times, right?”* – Participant 5 |
|  | *“I would say how can we contact you when we're ready to see you, will you be in the waiting room, can we text you, can we phone you, do you want your name on a board and we wave it around to everybody, whatever, let them choose.”* – Participant 5 |
|  | *“Just always offering people an option, a written option, because for people with hearing loss, having something written down, I mean texting has saved our lives, right?”* – Participant 2 |
|  | *“I think that if an organization has multiple approaches available, like say they might have a hearing induction loop at many points of the hospital … maybe they have a Pocketalker that can be borrowed to go around the hospital, perhaps they have some sort of device that can caption … it [addresses] multiple disabilities, different challenges. So I think it is really important that they take a multiple [approach].”* – Participant 10 |
|  | *“Captioning I'm sure is really great for people who only [have] hearing [loss] but for me somebody who [has vision loss] captioning doesn't work unless you make it size 500 and give me like 2 minutes to read one sentence, you know. It doesn't work for me, and I don't know how we make it more accessible for people who have also visually impaired.”* – Participant 8 |
| What works in one setting may not work in others | *“There is no way you are going to loop the entire hospital because that is not going to happen. A lot of times you're having surgery or having a room and having an examination and you have to take your hearing aids out or [cochlear implant] or something else off, so now you're totally deaf.”* – Participant 1 |
|  | *“There are portable hearing loops. Hearing induction loops generally can sit on the countertop and … there are models that actually have handles … and you just carry it around with you, so it's portable. There are also ones that can be hardwired in a counter hub so they would never move so there are many options. I think that the hospital should have them at the front desk or where people check in and stuff permanently but they should have ones that you should pick up and take to your appointment with your doctor, have your chat with the medical professional, and then you just return it to the front desk where you got it from afterwards. It is just like hard of hearing devices when you go to the movie, you pick it up when you get your ticket and you return it and they recharge it.”* – Participant 10 |
|  | *“I think it is also important to remember too though that we all have the luxury of having a speaking voice and not everybody with a disability has that opportunity, and so I also think that -- and I don't want to speak too much on this because I'm not somebody that is non-verbal and using adaptive technology so I'm not the best to speak on it, but I think that's another part of it, hospitals need to be more accepting and accommodating to adaptive technology as well in terms of people that are not speaking” –* Participant 8 |
|  | *“One of the things with hearing aids, no matter how good they are, is that you miss things. I’m on the phone, somebody calls me and I phone them and they say – what did you say? They are asking me, I said, I can't hear you.”* – Participant 12 |
|  | *“The thing about the [captioning] apps is they're not going to be able to know the name of medication or anything that is very specialized. So they become a lot less useful the more in depth the conversation is.”* – Participant 2 |
|  | *“If you have [a microphone] right in front of your mouth, they work great. If they are three feet away, for every three feet, you lose 50% of the power. So 50% of 50% of 50% pretty soon all you get is noise”* – Participant 3 |
