# Supplementary material for: Hospital care does not meet the communication needs of patients with hearing loss: A qualitative study of patient experiences
Source: PLoS One. 2025 Oct 10;20(10):e0333587. doi: 10.1371/journal.pone.0333587 (PMC12513649; doi:10.1371/journal.pone.0333587)
Supplement: Attachment — Submitted filename: PLOS ONE FEEDBACK CM.pdf [file pone.0333587.s003.pdf]

PLOS ONE

Hospital care does not meet the communication needs of patients with hearing loss: A qualitative study of patient experiences.

--Manuscript Draft—

Reviewer Feedback: Carmen Milton

Overview

This manuscript presents a qualitative study examining the communication experiences of patients with hearing loss during hospital stays in Alberta, Canada. The research uses focus groups and interviews, thematically analysed, to uncover systemic, provider, and patient-level barriers and highlight communication needs and potential strategies for improvement.

Assessment Against PLOS ONE Criteria

1. Originality and Fit

I found the study to be an original and much-needed contribution to the literature on hospital care for individuals with hearing loss. The use of qualitative methods, specifically focus groups and interviews, felt particularly effective for capturing the lived experiences of your participants. The approach aligns closely with the aims of PLOS ONE, as the findings not only explore a gap in current practice but also offer thoughtful pathways for improving care. The reporting of three core themes stemming from patient voices particularly stood out to me and reinforced the fit of the work within the journal's mission.

2. Prior Publication and Replication

I appreciated the clarity regarding the novelty of the data and findings. The researcher appropriately referenced existing literature and made it clear how their analysis adds new insight, rather than retreading ground already covered by previous studies. I did not encounter any sections or data that appeared previously published or duplicated, and the researchers situated their findings within the context of prior research without redundancy.

3. Technical Standards and Methodological Rigor

The methods section provided useful information on participant selection, demographic considerations, and the analytic process. I noted and valued the explicit mention of recruiting until "saturation of both demographics and participant experiences" was reached (lines 94–97). I especially appreciated how the researchers aimed for diversity across age, gender, place of residence, language, and socio-demographic variables. What I would still find helpful, for transparency, is a brief description of *how they determined* when saturation occurred. For example, did they discuss emerging themes at certain intervals? Was the decision consensus-based among the research team? Even a short clarification would help readers understand

the process. The systematic approach to coding and triangulation with two coders and member-checking also adds credibility to the work.

4. Conclusions and Interpretation

The conclusions are grounded in data and, in my view, are appropriately cautious. By basing suggestions for improvement on the themes that emerged (rather than overstating or generalising), the researchers demonstrate an effective and responsible use of qualitative data. The participant quotes interspersed throughout the results section really help connect the findings to real patient experiences. I appreciated the clear distinction between what the data can directly support and what remains speculative or worthy of future exploration.

5. Clarity and Quality of English

For the most part, I found the writing clear, organised, and accessible, with a straightforward academic tone. There are a few minor redundancies and repeated phrases, but none that significantly interrupt the flow. Cleaning up these small issues before final submission should be straightforward and will further polish what is already an accessible manuscript.

6. Ethics and Research Integrity

A full and transparent ethics statement is included (REB22-1818, University of Calgary Conjoint Health Research Ethics Board) and describes that all participants gave written informed consent. The financial disclosure and conflict of interest statements are also complete and clear, with the funding agency's non-involvement in key aspects of the study explicitly stated. Research integrity is further demonstrated through careful data handling and attention to confidentiality.

7. Reporting Guidelines and Data Availability

The methods and results are thoroughly reported, with particular attention to the qualitative approach. I recognise that the researchers cannot share their data publicly due to confidentiality concerns, which is a common and appropriate constraint for this type of sensitive health research. If possible, they may consider indicating whether data might be made available on a restricted basis to qualified researchers via their institution or a data access committee, but I do not see any deficiencies regarding reporting standards.

FEEDBACK TO AUTHORS

Thank you for sharing your manuscript, "Hospital care does not meet the communication needs of patients with hearing loss: A qualitative study of patient

experiences." I appreciate the opportunity to review such a thoughtful and timely contribution.

Strengths of the study

- Your study is a much-needed exploration of a topic that impacts many patients and their families, yet is rarely addressed in hospital policy or training.
- The qualitative approach is very well suited for the research questions and gives a clear, real-world voice to participants' needs and insights.
- The reporting is meticulous, with methods, recruitment, and analysis explained step-by-step and transparently.
- The use of direct quotes throughout the results brings authenticity and clarity.

Where You Can Improve

- I appreciate that you have addressed both demographic and experiential saturation and listed the key variables considered. If possible, adding a brief description of how you monitored for saturation (for example, indicating after which interviews or groups you judged that saturation was reached) would further strengthen clarity and transparency for the reader.
- Since participants reflected on hospital experiences from the past several years, you might clarify what steps, if any, were taken to reduce the possible influence of recall bias or memory gaps.
- The manuscript is clear overall, just a few minor issues with repeated wording and phrasing could be rephrased for smoother reading. For example, in the abstract, the phrase 'patients with hearing loss' is used repeatedly in close proximity: "Tools and strategies to facilitate two-way communication with patients with hearing loss and other members of their healthcare team may help address this gap. This study describes the communication-related experiences of patients with hearing loss in Alberta hospitals, which can help inform future strategies in this setting."

  Rewording for conciseness, perhaps by substituting with 'these patients' or streamlining sentences, would make for smoother reading.

  I also noticed similar patterns in the results and introduction, where closely related ideas are covered in multiple, slightly different ways. Consolidating or varying phrasing in these sections would further enhance clarity and flow.
- I encourage you to be mindful in your discussion section that practice and policy suggestions are framed as proposed improvements, not prescriptive recommendations. This preserves both the strength and boundaries of qualitative findings.

- You have clearly explained why data can't be shared openly. If feasible within your institution, stating whether access is possible for qualified researchers by request could further support transparency.

Thank you again for your dedication to equity in healthcare communication. This research will be invaluable for clinicians, administrators, and advocates seeking to improve hospital experiences for patients with hearing loss.

NOTE TO EDITOR

Recommendation: Minor Revision

This manuscript meets PLOS ONE's requirements for original research, ethics, methodological rigour, and clarity. The work provides an important addition to the literature and offers actionable pathways for improvement in hospital-based communication for people with hearing loss.

The study design and analytic approach are sound and clearly reported. The main limitations are acknowledged and appropriately contextualised. I have recommended only minor revisions: adding brief clarifications on data saturation and recall bias, a very light editorial review for typographic clarity, and ensuring all implications remain suggestions rather than directives.

Assuming these points are addressed, I strongly support publication. This study brings much-needed attention to communication equity in hospital care.
