# Supplementary material for: Hospital care does not meet the communication needs of patients with hearing loss: A qualitative study of patient experiences
Source: PLoS One. 2025 Oct 10;20(10):e0333587. doi: 10.1371/journal.pone.0333587 (PMC12513649; doi:10.1371/journal.pone.0333587)
Supplement: Attachment — Submitted filename: Table Recommendation.docx [file pone.0333587.s004.docx]

Table 1 recommended layout: (add additional columns as needed)

| Participant ID | Self-identified Gender | Age (Years) | Geographic Location |
| --- | --- | --- | --- |
| P01 |  |  |  |
| P02 |  |  |  |
| P03 |  |  |  |
| P04 |  |  |  |
| P05 |  |  |  |
| P06 |  |  |  |
| P07 |  |  |  |
| P08 |  |  |  |
| P09 |  |  |  |
| P10 |  |  |  |
| P11 |  |  |  |
| P12 |  |  |  |
| P13 |  |  |  |
| P14 |  |  |  |
